# Characterization of a multiprotein complex involved in excitation-transcription coupling of skeletal muscle

**DOI:** 10.1186/s13395-016-0087-5

**Published:** 2016-04-11

**Authors:** Manuel Arias-Calderón, Gonzalo Almarza, Alexis Díaz-Vegas, Ariel Contreras-Ferrat, Denisse Valladares, Mariana Casas, Héctor Toledo, Enrique Jaimovich, Sonja Buvinic

**Affiliations:** Centro de Estudios Moleculares de la Célula, Instituto de Ciencias Biomédicas, Facultad de Medicina, Universidad de Chile, Santiago, 8380453 Chile; Programa de Biología Molecular y Celular, Instituto de Ciencias Biomédicas, Facultad de Medicina, Universidad de Chile, Santiago, 8380453 Chile; Instituto de Investigación en Ciencias Odontológicas, Facultad de Odontología, Universidad de Chile, Sergio Livingstone Pohlhammer 943, 8380492 Santiago, Chile

**Keywords:** Multiprotein complex, Excitation-transcription coupling, Dihydropyridine receptor, Nucleotide receptors, Pannexin 1, Skeletal muscle plasticity

## Abstract

**Background:**

Electrical activity regulates the expression of skeletal muscle genes by a process known as “excitation-transcription” (E-T) coupling. We have demonstrated that release of adenosine 5′-triphosphate (ATP) during depolarization activates membrane P2X/P2Y receptors, being the fundamental mediators between electrical stimulation, slow intracellular calcium transients, and gene expression. We propose that this signaling pathway would require the proper coordination between the voltage sensor (dihydropyridine receptor, DHPR), pannexin 1 channels (Panx1, ATP release conduit), nucleotide receptors, and other signaling molecules. The goal of this study was to assess protein-protein interactions within the E-T machinery and to look for novel constituents in order to characterize the signaling complex.

**Methods:**

Newborn derived myotubes, adult fibers, or triad fractions from rat or mouse skeletal muscles were used. Co-immunoprecipitation, 2D blue native SDS/PAGE, confocal microscopy *z*-axis reconstruction, and proximity ligation assays were combined to assess the physical proximity of the putative complex interactors. An L6 cell line overexpressing Panx1 (L6-Panx1) was developed to study the influence of some of the complex interactors in modulation of gene expression.

**Results:**

Panx1, DHPR, P2Y_2_ receptor (P2Y_2_R), and dystrophin co-immunoprecipitated in the different preparations assessed. 2D blue native SDS/PAGE showed that DHPR, Panx1, P2Y_2_R and caveolin-3 (Cav3) belong to the same multiprotein complex. We observed co-localization and protein-protein proximity between DHPR, Panx1, P2Y_2_R, and Cav3 in adult fibers and in the L6-Panx1 cell line. We found a very restricted location of Panx1 and Cav3 in a putative T-tubule zone near the sarcolemma, while DHPR was highly expressed all along the transverse (T)-tubule. By Panx1 overexpression, extracellular ATP levels were increased both at rest and after electrical stimulation. Basal mRNA levels of the early gene *cfos* and the oxidative metabolism markers citrate synthase and peroxisome proliferator-activated receptor gamma coactivator 1-alpha (PGC1α) were significantly increased by Panx1 overexpression. Interleukin 6 expression evoked by 20-Hz electrical stimulation (270 pulses, 0.3 ms each) was also significantly upregulated in L6-Panx1 cells.

**Conclusions:**

We propose the existence of a relevant multiprotein complex that coordinates events involved in E-T coupling. Unveiling the molecular actors involved in the regulation of gene expression will contribute to the understanding and treatment of skeletal muscle disorders due to wrong-expressed proteins, as well as to improve skeletal muscle performance.

## Background

The gene expression regulation is a crucial event for skeletal muscle that determines the “muscle plasticity” that allows muscle transformation and remodeling to adapt to environmental demands [1]. It has long been known that exercise drives gene expression in muscle cells at the level of structural proteins and energetic metabolism enzymes [2–4] and that innervation is essential to keep muscle integrity and function [5–8]. Until now, regulation of gene expression in muscle has been attributed to mechanical, hormonal, or metabolic changes and probably all these factors contribute to the complex response to exercise, but very little attention has been directed to the action potential in the muscle fiber as a regulator of this process. We have evidence that an important number of genes are regulated this way, via the “excitation-transcription” (E-T) coupling process, as detailed below.

We have previously described that membrane depolarization of skeletal myotubes evokes a fast calcium transient during the stimuli that promotes a contractile response through “excitation-contraction coupling” (E-C coupling) and a slow calcium transient peaking 60–100 s later, mostly associated to cell nuclei [9–13]. Slow Ca^2+^ transients are involved in the E-T coupling mechanism, which relates membrane depolarization with gene expression [9, 13–16]. The signaling pathway begins at the voltage sensor dihydropyridine receptor (DHPR, Ca_v_1.1), which by a mechanism involving G protein [17], activates PI3 kinase and phospholipase-C (PLC) to produce inositol 1,4,5-trisphosphate (IP_3_) that diffuses to the cytosol and reaches IP_3_ receptors (IP_3_Rs) located both at the sarcoplasmic reticulum (SR) membrane and at the nuclear envelope, promoting Ca^2+^ release [9]. IP_3_-mediated Ca^2+^ signals induce both a transient activation of ERK and transcription factor CREB and an increase in early genes *c-fos, c-jun*, and *egr-1* mRNA levels and in late genes such as *troponin I*, *interleukin 6* (*IL-6*), *Hmox*, *and Hsp-70* mRNA levels after depolarization of normal skeletal muscle cells [14–16, 18]. These evidences link slow calcium transients with possible trophic effects of nerve activity and could shed light on the process of muscle cell E-T coupling and plasticity.

In the past years, we have demonstrated that adenosine 5′-triphosphate (ATP) is a relevant mediator between membrane depolarization, calcium signaling, and gene expression, in both skeletal primary cultures and adult fibers [19, 20]. Our studies place extracellular ATP as a master regulator of the E-T coupling leading to muscle plasticity (as reviewed in [21]). We demonstrated that ATP is released by physiological activity of skeletal muscle cells through pannexin 1 (Panx1) channels. ATP and its metabolites activate P2Y/P2X receptors, increasing intracellular calcium levels that modulate the expression of immediate early genes (*cfos*), exercise-induced genes (*IL-6*), or plasticity-related genes (*troponin I fast/slow*) [19–23]. We have recently demonstrated for the first time that this molecular mechanism allows adult muscle fibers to recognize different frequencies and translate them in different gene expression profiles [20]. We can conclude that the released ATP and its metabolites are fundamental mediators between electrical stimulation, slow intracellular calcium transients, and gene expression related to muscle plasticity [19]. Taken all the previous work together, we consider that gene expression evoked by sarcolemma depolarization would require the proper coordination between the voltage sensor DHPR, Panx1 channel as the ATP-release conduit, P2X/P2Y nucleotide receptors, and several intracellular molecules involved in signal transduction.

In the post-genomic era, the knowledge of the expression profile of a single protein is not enough to understand biological processes. Cell signaling is mainly regulated by “multiprotein complexes”, a cluster of proteins associated to achieve coordinated steps in a transduction pathway [24–28]. How such a complex assemble/disassemble or changes its composition after specific cell stimulation (“signaling complex”) or its alteration in health and disease is an imperative question in the study of cell function. Several signaling complexes have been described in cardiac and skeletal muscles. In cardiac muscle, the requirement of macromolecular complexes for β-adrenergic modulation of a potassium channel [29], for the proper expression and activity of the sodium channel Nav1.5 [30] and for determining cardiac phenotype [28] has been demonstrated. Assembly of signaling complexes between different voltage dependent calcium channels and G protein coupled receptors was reviewed by Altier [31]. A macromolecular E-C coupling complex has been proposed in cardiac and skeletal muscles, organizing RyR, Ca_v_1.1 channel α1S-subunit (in skeletal muscle), calmodulin, calstabin, A-kinase anchor protein (AKAP), protein kinase A (PKA), Ca^2+^/calmodulin-dependent protein kinase II (CaMKII), sorcin, calsequestrin, triadin, and junctin (reviewed in [32–34]). In skeletal muscle, a multiprotein complex between the store operated calcium channel, transient receptor potential channel 1 (TRPC1), dystrophin (Dys), and syntrophin has been established, and its disassembly was proposed as a putative explanation for calcium alterations observed in dystrophic muscle cells [35].

In the present work, we demonstrated, using several experimental approaches, the existence of a multiprotein complex that harbors several proteins previously related to the E-T coupling process, such as DHPR, Panx1, and P2Y_2_ receptor (P2Y_2_R). We also detected Dys and caveolin-3 (Cav3), two proteins with relevant roles in scaffolding and signaling, as part of this complex. We also demonstrated that upon overexpressing one of the complex constituents (Panx1), both ATP release and gene expression were potentiated. We have therefore begun the characterization of a multiprotein complex relevant to the E-T coupling process in muscle cells.

## Methods

### Reagents

Dulbecco’s modified Eagle’s medium, Minimal Essential Medium Eagle alpha modification (α-MEM), neomycin, fetal bovine serum, horse serum, and penicillin-streptomycin were obtained from Thermo Fischer Scientific (Waltham, MA, USA). Collagenase type II was from Worthington Biochemical (Lakewood, NJ, USA). Complete™ Mini protease inhibitors were from Roche Applied Science (Indianapolis, IN, USA). Protein A/G agarose was from Santa Cruz Biotechnology (Santa Cruz, CA, USA). Mouse anti-Strep antibody was from IBA GmbH (Gottingen, Germany), anti-caveolin-3 was from BD Bioscience (San José, CA, USA), and anti-Dys was from Leica Biosystems (Wetzlar, Germany). Rabbit anti-P2Y_2_R and anti-Panx1 antibodies, mouse anti-DHPR α-1 antibody, and secondary anti-mouse and anti-rabbit antibodies conjugated to either horseradish peroxidase or Alexa Fluor® dyes were from Thermo Fischer Scientific. Enhanced chemiluminescence (ECL) reagents were from Amersham Biosciences (Piscataway, NJ, USA). ECM (gel from Engelbreth-Holm-Swarm murine sarcoma) matrix was from Sigma-Aldrich (St. Louis, MO, USA). All other reagents used were of analytical quality.

### Construction of the plasmid DNA for expressing recombinant Panx1-Strep-tag protein fusion

Panx1 complementary DNA (cDNA) was subcloned from the pCMV-SPORT6 (Open Biosystems, Dharmacon GE Healthcare, CO, USA) to pEXPR-IBA103 vector (IBA GmbH, Goettingen, Germany), to obtain the DNA for the Panx1 protein fused to the Twin-Strep-tag at the C terminus (pEXPR- IBA103-Panx1). Specific cDNA was amplified by PCR, adding BsaI restriction sites for the cloning in-frame with the Twin-Strep-tag epitope. The proper cloned sequence was confirmed by sequencing (Center for automated sequencing, Pontificia Universidad Católica, Chile).

### L6-Panx1 cell line establishment

L6 myoblasts at 60 % confluence were transfected with either pEXPR-IBA103 or pEXPR-IBA103-Panx1, by using Lipofectamine 2000 (Life Technologies, NY, USA). After 48 h, the cells were incubated with 1.6 mg ml^−1^ neomycin and maintained for 2 weeks. Clones were isolated using cloning cylinders, and Panx1-Strep expression was assessed by immunocytochemistry using anti-Strep antibodies. Clones with more than 80 % expression were selected. Positive clones were then maintained with 0.8 mg ml^−1^ neomycin.

### Cell culture

L6-Panx1 myoblasts were maintained in α-MEM supplemented with 10 % fetal bovine serum in a humidified atmosphere containing 5 % CO_2_ and 95 % air at 37 °C. To induce differentiation into myotubes, α-MEM supplemented with 2 % fetal bovine serum was used for 7–8 days.

### Isolation of adult fibers

Six- to 8-week-old mice were used throughout this work. All protocols were approved by the Bioethics Committee, Faculty of Medicine, Universidad de Chile. Isolated fibers from the *flexor digitorum brevis* (FDB) muscle were obtained by enzymatic digestion with collagenase type II (90 min with 400 U ml^−1^) and mechanic dissociation with fire-polished Pasteur pipettes, as previously described [36]. The isolated fibers were seeded in ECM-coated dishes and used 20 h after seeding.

### Skeletal muscle derived triad-enriched fractions

Preparation of triad-enriched fractions from the back and hind leg muscles derived from 6- to 8-week-old BalbC mice were performed as previously standardized in our laboratory for frog and rabbit muscles, using differential centrifugation [37–39].

### ATP detection using luciferin/luciferase assay

Fifty microliters of extracellular sample was added to 20 μl of CellTiter-Glo® luminescent cell viability assay (Promega, Madison, WI), and the detection of ATP was develop as previously described [19]. Briefly, the samples were quantified in a luminometer, and the readings were interpolated in a standard curve (100 fmol to 10 pmol of ATP) to detect the ATP concentration under each condition. After sample collection, cells were lysed and the total protein amount was measured. Data were calculated as pmol extracellular ATP/mg total protein.

### Co-immunoprecipitation and Immunoblot

Triad-enriched fractions (100 μg protein) were solubilized for 1 h in 200 μl of lysis buffer (20 mM Tris-HCl pH 7.4, 0.1 % Nonidet P-40, 5 mM EDTA pH 8, 10 mM EGTA pH 7.8, 140 mM NaCl, 10 % glycerol, and protease inhibitors). A 20-min 15,000-g supernatant fraction was incubated 30 min with 10 μg A/G agarose as a pre-clearing strategy. The beads were spun down by centrifugation and washed three times with 200 μl of washing buffer (25 mM HEPES pH 7.5, 0.2 % Nonidet P-40, 140 mM NaCl, 0.1 % BSA, 10 % glycerol, and protease inhibitors). After the pre-clearing step, the whole cell extracts were incubated for 4 h with the correspondent antibody and then incubated 30 min with 50 μg A/G agarose beads. The beads pellet was washed three times with washing buffer. The whole sample obtained after this step was resolved by SDS-PAGE in 7–10 % gels, transferred to polyvinylidene difluoride (PVDF) membranes and blotted with the corresponding antibody.

For each immunoprecipitation assay, positive controls of protein expression at the whole lysate before co-IP and negative controls of pre-clearing assays (fraction of the lysate that precipitates with the A/G beads in the absence of antibodies) were performed.

### 2D blue native SDS-PAGE

The triad samples were analyzed by two-dimensional blue native SDS-PAGE (BN/SDS-PAGE) according to the method of Wittig et al. [40]. Briefly, a stacking gel of 3 % and a separating gradient gel of 3–8 % were used. One hundred micrograms of triads was solubilized with 30 μl of solubilization buffer (50 mM Imidazol, 500 mM 6-aminohexanoic acid, 1 mM EDTA, pH 7.0) adding 5 μl of 20 % Digitonin (*w*/*v*). After solubilization, the sample was centrifuged 25 min at 17,800 g at 4 °C. Then, Coomassie Blue was added to achieve a ratio of detergent/Cooomassie of 8 g/g. 1D BN-PAGE electrophoresis was conducted at 100 V for 2 h at 4 °C, using anode buffer (25 mM Imidazole pH 7) and cathode B buffer (50 mM Tricine, 7.5 mM Imidazole, 0.02 % Coomassie Blue, pH 7). The cathode B buffer was replaced for the cathode buffer B/10 (50 mM Tricine, 7.5 mM Imidazole, 0.002 % Coomassie Blue, pH 7) and conducted at 100 V for 4 h. After 1D electrophoresis, the lane was cut and incubated with 2-mercaptoethanol for 30 min and the second dimension was resolved by SDS-PAGE in 3–8 % gels. Proteins were transferred to the PVDF membranes (40 V, overnight (ON) at 4 °C) and blotted with the corresponding antibody.

### Protein proximity assays and Immunofluorescence

Protein-protein proximity assays were detected in situ using the Duolink II red starter kit (Olink Bioscience, Uppsala, Sweden) according to the manufacturer’s instructions. Briefly, primary antibodies against selected proteins (one raised in mouse and one raised in rabbit) were applied ON at 4 °C in a humid chamber. Duolink plus and minus secondary antibodies against the primary antibodies were then incubated for 1 h at 37 °C. These secondary antibodies were provided as conjugates to oligonucleotides that were tied together in a closed circle by Duolink Ligation Solution, if the antibodies were in close proximity (<40 nm). Finally, polymerase was added to amplify any existing closed circles, and detection was achieved with complementary fluorescently labeled oligonucleotides. As a negative control, we performed a proximity ligation assay (PLA) between two proteins known not to interact, as Lamina-associated polypeptide 2 (Lap2) and DHPR.

To unveil specific location of PLA-dots on the surface or in the depth of the cell, we compared staining of the whole cell z-stacks collapsed in a single image (30–35 slices, 1 μm each) with the collapse of the six middle stacks (1 μm each), using ImageJ software (NIH).

To perform immunofluorescence experiments and z-projection reconstruction for the whole fiber, cells were rinsed with ice-cold PBS and fixed for 40 min with 4 % paraformaldehyde (Electron Microscopy Science, Hatfield, PA, USA) in PBS. The cells were rinsed with ice-cold PBS and incubated with 100 mM glycine in PBS for 10 min. Then, the fibers were permeabilized with 0.1 % Triton X-100 in PBS and blocked with 4 % BSA. The cells were incubated with primary antibodies ON at 4 °C. Finally, the cells were washed three times with PBS for 5 min each and incubated with anti-mouse and anti-rabbit Alexa 488/Alexa 546 as appropriate. The samples were mounted in Dako anti-fading reactive (Dako, Denmark) and stored at 4 °C until use. All images were acquired with a Carl Zeiss Axiovert 135 M Laser Scanning Microscope, with an Apo Plan 63x, NA 1.4 objective. Images deconvolution and processing were performed using ImageJ software (NIH). Co-localization analysis of immunofluorescences and z-projection reconstructions were assessed by Manders’ coefficients [41]. Three region of interest (ROI) were evaluated per image for the Manders’ coefficient quantification, using the JACoP pluggin of ImageJ software (NIH).

### Quantitative PCR

Total RNA was isolated from skeletal L6 cells using Trizol reagent (Invitrogen, Corp., Carlsbad, CA, USA) according to manufacturer’s protocol. cDNA was prepared from 1 μg RNA, using SuperScript II enzyme (Invitrogen), according to the manufacturer’s protocol. Quantitative real-time PCR (qPCR) was performed using Fast SYBR® Green Master Mix and the StepOne™ Real-Time PCR System from Thermo Fischer Scientific (Waltham, MA, USA). The primers used were as follows: Panx1: CGATCGTGGAGCAGTACTTGAAGA (sense), AGGAGAGGCTGAAGTAGTAGCT (antisense); Panx1-Strep-tag: AACTCCCATGTCTGCAGAGAT (sense), GAACCTCCACCTTTCTCGAA (antisense); IL6: CCAATTTCCAATGCTCTCCT (sense), ACCACAGTGAGGAATGTCCA (antisense); cfos: AGGCCGACTCCCTTCTCCAG (sense), CAGATAGCTGCTCTACTTTGC (antisense); citrate synthase: TGCTGGGGGTCTCCCTGTCC (sense), TGGGACCAGGCCCGAAGAGG (antisense); peroxisome proliferator-activated receptor gamma coactivator 1-alpha (PGC1α): TGATGTGAATGACTTGGATACAGACA (sense), GCTCATTGTTGTACTGGTTGGATATG (antisense); glyceraldehyde 3-phosphate dehydrogenase (GAPDH): CAACTTTGGCATTGTGGAAG (sense), CTGCTTCACCACCTTCTTG (antisense). All primers used presented optimal amplification efficiency (between 90 and 110 %). PCR amplification of the housekeeping gene GAPDH was performed as a control. Thermocycling conditions were as follow: 95 °C for 20 s previous to 40 cycles of 95 °C for 3 s and 60 °C for 30 s. Expression values were normalized to GAPDH and are reported in units of 2^−ΔΔCT^ ± standard error of the mean (SEM) as described [42]. CT value was determined by StepOne software when fluorescence was 25 % higher than background. PCR products were verified by melting-curve analysis.

### Statistical analysis

Data of *n* experiments were expressed as mean ± SEM. The significance of differences among conditions was evaluated using Student’s *t* test for unpaired data or two-way ANOVA followed by Tukey post hoc test for multiple comparisons. A *p* value <0.05 was considered statistically significant.

## Results

### DHPR co-precipitates with Panx1, P2Y_2_R, and dystrophin in murine adult muscle triad preparations

**Fig. 1:**
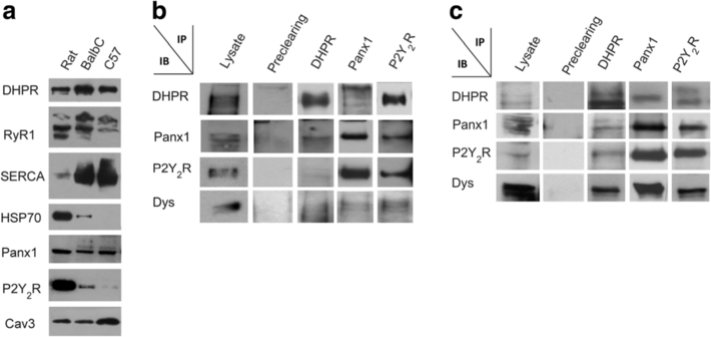
Co-immunoprecipitation evidences for a multiprotein complex involved in excitation-transcription coupling. **a** Characterization of triad-enriched fractions derived from rat or mouse muscles. Several proteins were detected by immunoblot: dihydropyridine receptor (DHPR), ryanodine receptor 1 (RyR1), sarco/endoplasmic reticulum Ca^2+^-ATPase (SERCA), heat shock protein 70 (HSP70), pannexin 1 (Panx1), P2Y_2_ receptor (P2Y_2_R) and caveolin-3 (Cav3). **b**, **c** Several protein components for the excitation-transcription machinery co-precipitate both in mouse adult muscle triad preparation (**b**) and in rat myotube extracts (**c**). The voltage sensor (DHPR), the ATP-release channel (Panx1), and a receptor for extracellular ATP (P2Y_2_R) co-precipitate suggesting a multiprotein complex. The scaffold protein dystrophin (Dys) co-precipitate with DHPR, Panx1, and P2Y_2_R. Protein immunodetection in the whole lysate and pre-clearing samples are shown as positive and negative controls, respectively (**b**, **c**)

Enriched triad preparations derived from the back and hind leg muscles of an adult rat (Wistar) or mouse (BalbC or C57) were obtained and characterized. Expression of the transverse (T)-tubule marker DHPR was detected, as well as the sarcoplasmic reticulum markers ryanodine receptor 1 (RyR1) and sarco/endoplasmic reticulum Ca^2+^-ATPase (SERCA) (Fig. 1a). Panx1 and P2Y_2_R, proteins putatively associated to DHPR, were also detected in triad preparations (Fig. 1a). Cav3, a protein related to scaffold and signaling, was also identified in these preparations (Fig. 1a). The mitochondrial resident protein Hsp70 was detected mainly in the rat triad preparation but was almost undetectable in mouse triads, suggesting that the latter was a cleaner fraction.

DHPR, Panx1, and P2Y_2_R co-immunoprecipitated with each other, and together with the scaffold and signaling protein Dys, both in triad preparations derived from adult BalbC back and hind limb skeletal muscles (Fig. 1b) and in cell extracts from newborn-derived rat myotubes (Fig. 1c). Positive controls of protein expression at the whole lysate before co-IP and negative controls of pre-clearing assays are shown (Fig. 1b, c).

### BN/SDS-PAGE assays reveal a multiprotein complex in skeletal triads, including DHPR, Panx1, P2Y_2_R, and Cav3

**Fig. 2:**
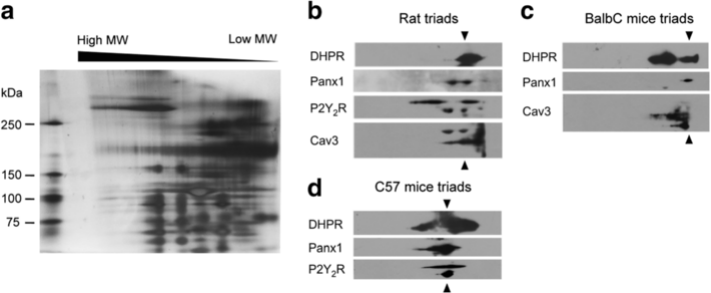
Isolation of protein complexes containing DHPR, P2Y_2_R, and Panx1 from skeletal triads using 2D blue native SDS-PAGE. **a** Silver staining of a mouse triad sample resolved by BN/SDS-PAGE. Several multiprotein complexes are observed as *dots* in a *same vertical lane*. **b**–**d** Immunoblot for complex putative interactors in triad-enriched samples derived from rat (**b**), BalbC mouse (**c**), or C57 mouse (**d**) previously resolved by BN/SDS-PAGE. Co-localization in the *same vertical lane* (as marked with *arrowheads*) suggests a multiprotein complex containing DHPR, Panx1, P2Y_2_R, and Cav3

Considering co-immunoprecipitation limitations, such as the whole cell lysate and solubilization that could allow unreal interactions, we performed a second approach using the BN/SDS-PAGE methodology. This technique has been described for one-step isolation of protein complexes from biological membranes, using highly controlled detergent concentrations [40]. We standardized detergent type and detergent/protein ratio to obtain a proper complex solubilization without complex disassembly, looking for a well-detectable signal of DHPR at high molecular weight (>600 kDa) at the non-denaturant first dimension. We tested Triton X-100, NP-40, and digitonin at three different detergent/protein ratios (2.5 – 5 – 10 g detergent/g protein), finding better results with digitonin at 5 g/g protein (not shown). We separated whole protein complexes by size in a native first dimension of blue native acrylamide gels. Each lane was then reduced in 2-mercaptoethanol and transferred to an SDS-PAGE second dimension, allowing each complex to separate its constituents in a same vertical line. Silver staining of the second dimension shows a pattern of dots organized in several vertical lines, suggesting co-existence of different multiprotein complexes in the triad preparation (Fig. 2a). By immunoblot of the second dimension with selected antibodies, we demonstrated that DHPR and Panx1 share a same vertical lane in triad samples of both rat and mouse (Fig. 2b–d). P2Y_2_R co-localized in the same vertical line with DHPR and Panx1 in both rat and c57 mouse triads (Fig. 2b, d). Cav3 shares a vertical line with DHPR and Panx1 in both rat and BalbC mouse triads (Fig. 2b, c). Altogether, these results suggest the assembly of a multiprotein complex containing DHPR, Panx1, P2Y_2_R, and Cav3 in rat and mouse skeletal muscles.

### Co-localization of DHPR, Panx1, P2Y_2_R, and Cav3 in adult skeletal fibers; immunofluorescence evidences

**Fig. 3:**
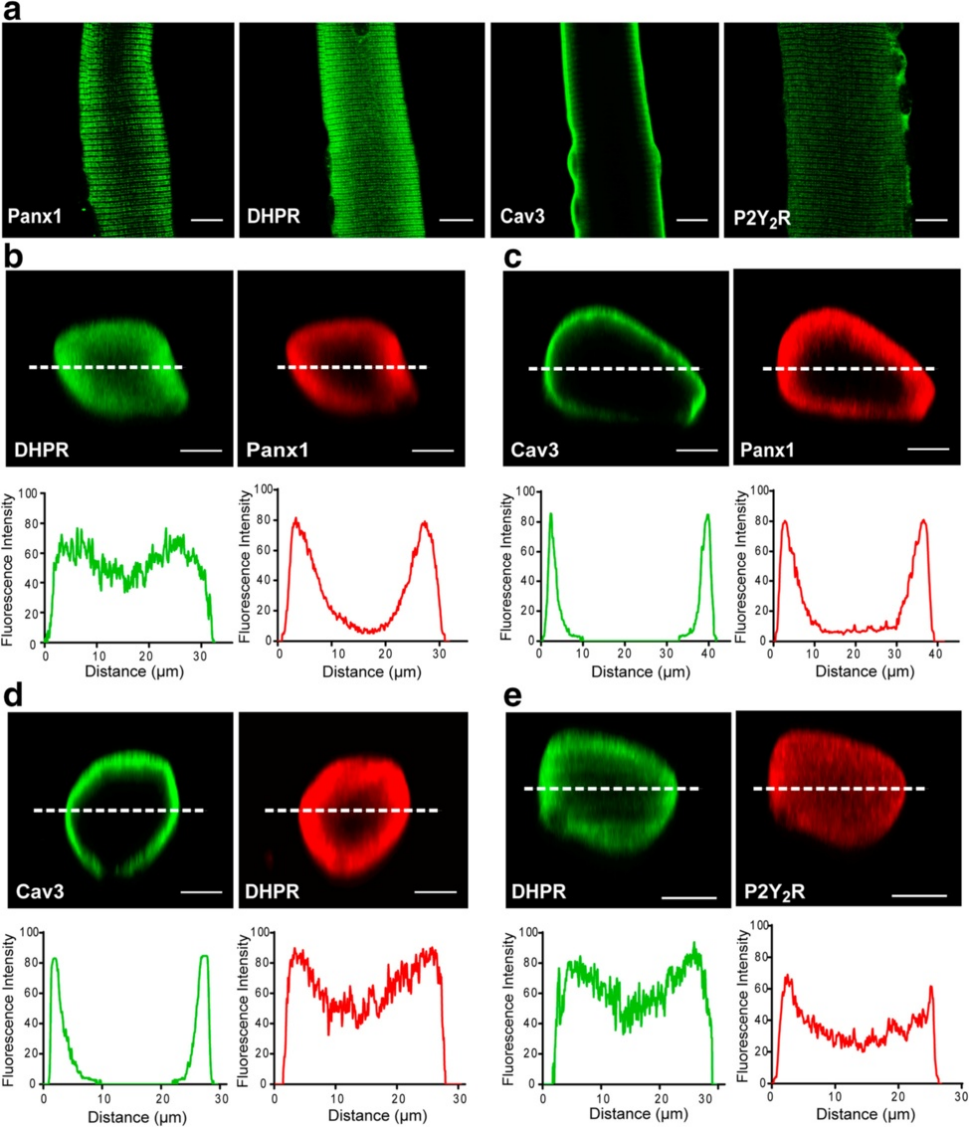
Subcellular distribution of DHPR, P2Y_2_R, Panx1, and Cav3 in isolated skeletal fibers. **a** Representative confocal immunofluorescence images for putative complex constituents in mouse *FDB*-isolated fibers. **b–e** Double-immunostaining and co-localization assays for different protein complex constituents. Protein distribution at the fiber *z*-axis was performed by line scan and z-projection reconstruction. Quantitation of fluorescence intensity in the area marked with a *dashed line* is shown under each image. *Scale bars* = 10 μm

In order to study cellular distribution of multiprotein complex constituents, we performed immunofluorescence in isolated FDB fibers. DHPR, Panx1, and P2Y_2_R showed a striated staining pattern (Fig. 3a). Cav3 was detected mainly at the sarcolemma, with little but distinctive presence in striations near the border (Fig. 3a). We analyzed a z-projection of the whole fiber and the correspondent fluorescence profiles of the median plane of the fiber (Fig. 3b–e). DHPR showed a staining pattern all across the fiber, remaining highly expressed down to the fiber center (Fig. 3b, d, e). Panx1 location was not homogeneous, with expression peaks within the first 10 μm from the cell surface and no stain at the fiber center (Fig. 3b, c). Similar to DHPR, P2Y_2_R staining was more homogeneous, with a strong stain at the cell surface that was only slightly reduced towards the fiber center (Fig. 3e). Cav3 showed thinner distribution peaks, highly concentrated at the cell surface, with no staining beyond 7 μm (Fig. 3c, d). These results show that the first 7-μm deep exhibits co-expression of DHPR, Panx1, P2Y_2_R, and Cav3, whose maximal expression occurs at 3–4-μm depth. In the zone of maximal expression pattern, a high co-localization was also observed between these proteins, as demonstrated by Manders’ coefficient analysis with co-localization values of 0.65 ± 0.08 (Panx1/DHPR), 0.71 ± 0.03 (Cav3/DHPR), 0.84 ± 0.05 (DHPR/P2Y_2_R), and 0.60 ± 0.02 (Cav3/Panx1).

**Fig. 4:**
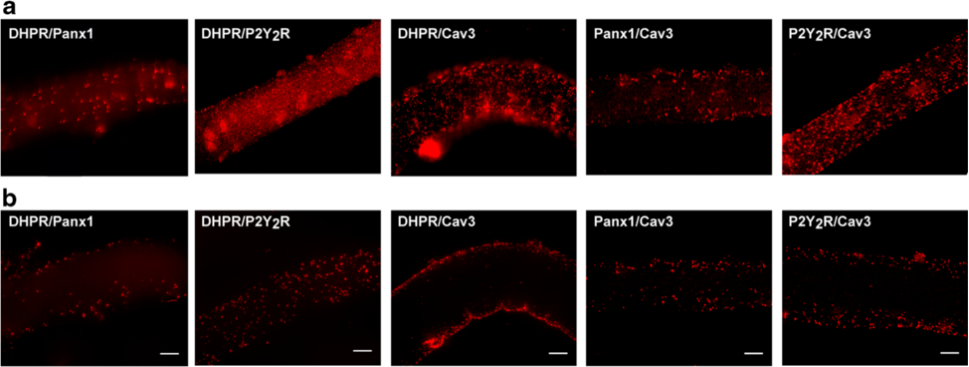
Association between DHPR, P2Y_2_R, Panx1, and Cav3 demonstrated by proximity ligation assay technology. **a** Protein-protein proximity was demonstrated using the PLA probe technology in a whole fiber, as indicated (DHPR with Panx1, DHPR with P2Y_2_R, DHPR with Cav3, Panx1 with Cav3, and P2Y_2_R with Cav3). Z-stacks were collected by confocal microscopy (35–40 slices, 1 μm each), and then pixels in all the stacks were collapsed into one single illuminated image. **b** Positive PLA dot distribution in the median plane of the fibers for the protein-protein proximity assessed (DHPR with Panx1, DHPR with P2Y_2_R, DHPR with Cav3, Panx1 with Cav3, and P2Y_2_R with Cav3). Images correspond to the six medial slices collapsed in a single image. *Scale bar =* 10 um

As a stronger tool for detecting protein-protein interaction at the whole skeletal muscle fiber, we used the *in situ* PLA, which shows proximity as fluorescence dots when the two proteins assessed are closer than 40 nm. Strong staining was detected between DHPR-Panx1, DHPR-P2Y_2_R, DHPR-Cav3, Panx1-Cav3, and P2Y_2_R-Cav3, in the whole fiber z-projection (Fig. 4a). Interestingly, the distribution pattern of the positive PLA dot in the medial plane of the muscle fiber showed a restricted co-localization area, near the sarcolemma, for DHPR-Panx1, DHPR-Cav3, Panx1-Cav3 and P2Y_2_R-Cav3 (Fig. 4b). In contrast, PLA assay for DHPR-P2Y_2_R showed a more homogeneous positive PLA-dot distribution through the fiber (Fig. 4b).

### Multiprotein complex is properly assembled in an L6 cell line overexpressing Panx1

**Fig. 5:**
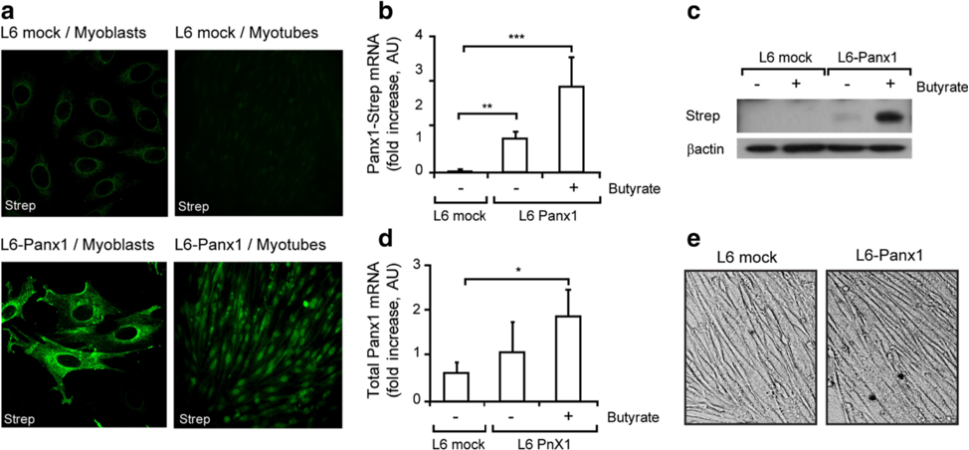
Characterization of an L6 skeletal cell line overexpressing Panx1-Strep-tag. **a** Representative immunofluorescence images of recombinant Panx1 overexpression, either in myoblast or myotube differentiation stages, using a Strep-tag antibody. Overexpression was induced with 5 mM sodium butyrate ON. **b** Panx1-Strep-tag mRNA levels were analyzed by qPCR using primers against the Strep epitope (*n* = 3). **c** Panx1 protein expression was assessed by immunoblot using Strep-tag antibodies. **d** The mRNA level of whole Panx1 (both endogenous and Panx1-strep) was analyzed by qPCR using primers against Panx1 (*n* = 3). **e** L6 cells overexpressing Panx1-Strep-tag properly differentiate to myotubes. Representative images of light microscopy are shown. In **b** and **d**, values are expressed as mean ± SEM. *p < 0.05; **p < 0.01; ***p < 0.001

In order to study the physiological events promoted by deregulation of the expression of one of the multiprotein complex interactors, we overexpressed Panx1 in the L6 rat myogenic cell line. We constructed a cDNA for fused Panx1 with Twin-Strep-tag at the C terminus and generated an L6 cell line stably expressing this protein (L6-Panx1). Overexpression was evoked using 5 mM sodium butyrate overnight. By immunofluorescence and confocal microscopy using an anti-Strep antibody, we demonstrated that Panx1-Strep was highly expressed in L6-Panx1 cells, both at the myoblast and the myotube differentiation stages (Fig. 5a, bottom panels). Staining was detected both at intracellular compartments and at the cell surface. In L6 mock cells, immunofluorescence for the Strep-tag only detected a weak and diffuse intracellular pattern, corresponding to the epitope expression (Fig. 5a, upper panels). Expression of the Panx1-Strep in L6-Panx1 cells and overexpression evoked after sodium butyrate treatment was demonstrated by qPCR (Fig. 5b) and by immunoblot (Fig. 5c), using primers or antibodies for the Strep-tag, respectively. Using Panx1 primers, which detect both endogenous Panx1 and exogenous Panx1-Strep-tag transcripts, we demonstrated that total Panx1 mRNA levels increased threefold by overexpression of the Panx1-Strep-tag, comparing L6-Panx1 cells with butyrate respect to the L6 mock cells (Fig. 5d). Treatment of L6 mock cells with sodium butyrate did not modify the total Panx1 mRNA levels (not shown). Furthermore, we demonstrated that Panx1 overexpression did not alter L6 differentiation to myotubes; both L6 mock and L6-Panx1 myotubes had similar morphology (Fig. 5e), and the differentiation process took 7–8 days for both cell lines.

**Fig. 6:**
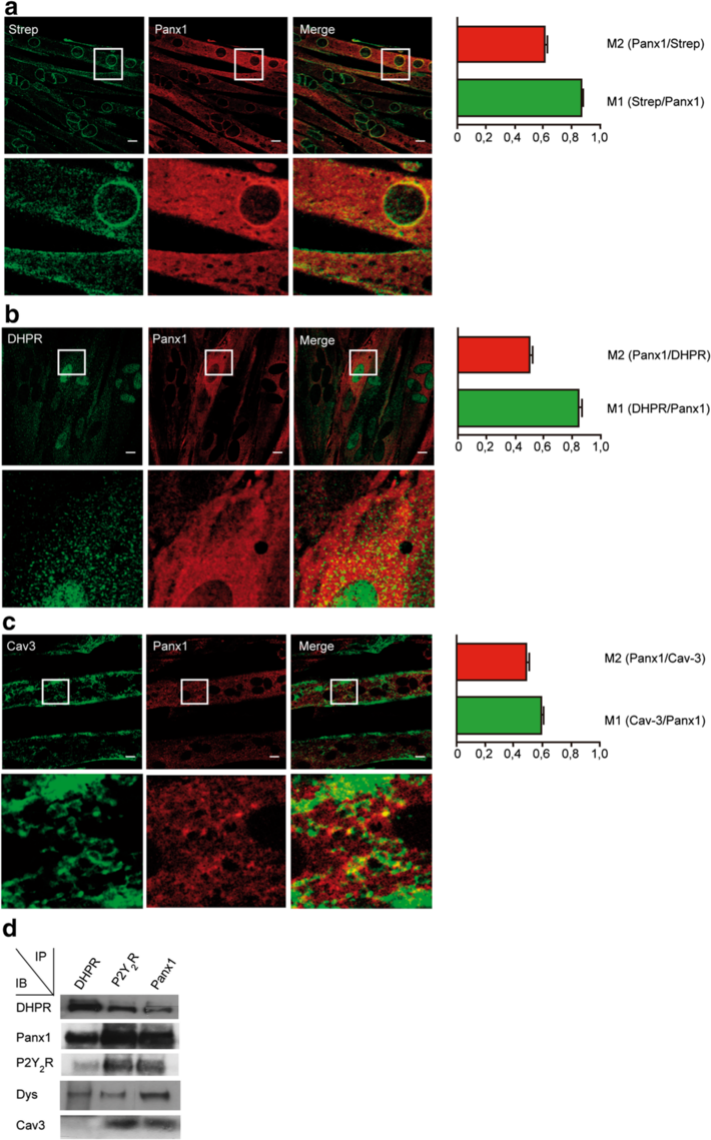
Multiprotein complex is properly assembled in L6 myotubes overexpressing Panx1. Double-staining immunofluorescence was performed using two different primary antibodies in the same sample, as detailed: anti-Strep/anti-Panx1 (**a**), anti-DHPR/anti-Panx1 (**b**), and anti-Cav3/anti-Panx1 (**c**). *Bar graphs* next to the image panels show Manders’ coefficients for the co-localization analysis. *Scale bar* = 10 μm. *White boxes* indicate area that is then magnified in the *row below. Bars* correspond to the average of the data obtained from three independent experiments, where three regions of interest (ROI) were evaluated per image for the Manders’ coefficient quantification. **d** Co-immunoprecipitation assay was performed in L6-Panx1 myotubes. Anti-Panx1, anti-DHPR, and anti-P2Y_2_R antibodies were used to immunoprecipitate whole lysates from L6-Panx1 cells. Panx1, DHPR, and P2Y_2_R all co-immunoprecipitated in these cells together with Dys and Cav3. Representative blot of the three experiments is shown

**Fig. 7:**
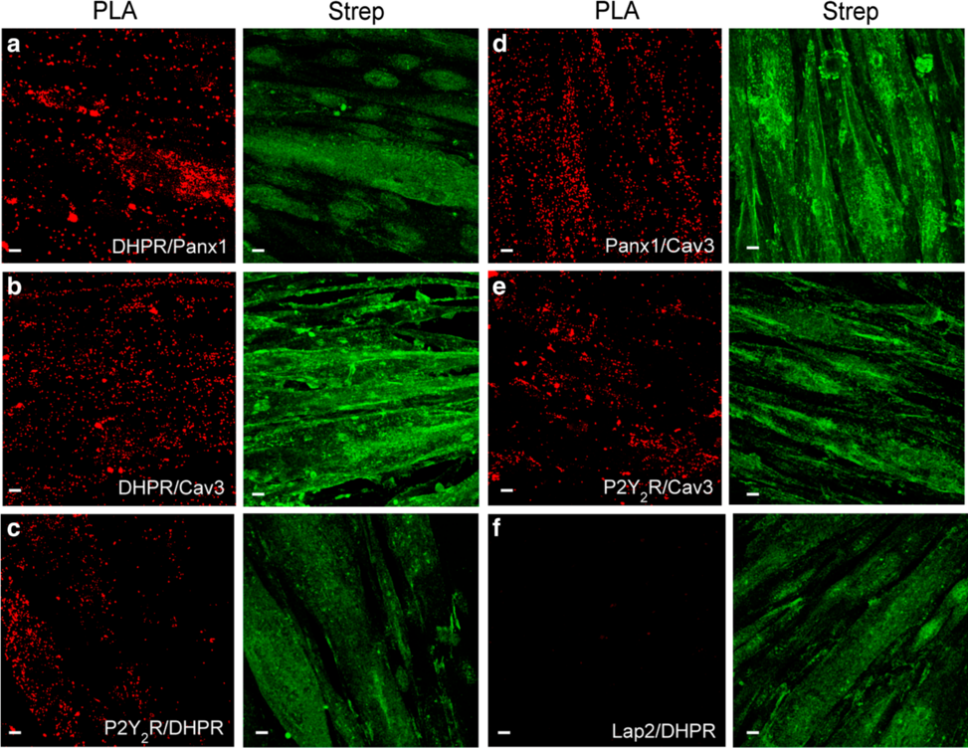
Nearness between DHPR, Panx1, P2Y_2_R, and Cav3 reinforced by proximity ligation assay technology. In situ proximity ligation assay (PLA) probes shown in *red* a well-defined closeness between DHPR and Panx1 (**a**), DHPR and Cav3 (**b**), DHPR and P2Y_2_R (**c**), Panx1 and Cav3 (**d**), and P2Y_2_R and Cav3 (**e**) in L6-Panx1 myotubes. No interaction was observed between DHPR and the nuclear envelope protein Lap2, as expected (**f**). To visualize cell limits and morphology, exogenous Panx1-Strep was stained in the same preparations using an anti-strep antibody (*green*). *Scale bars* = 10 μm

To assess the multiprotein complex assembly in L6-Panx1 cells, we used immunofluorescence followed by confocal microscope analysis or co-immunoprecipitation (Fig. 6). Confocal image analysis revealed a high co-localization index for Panx1-Strep stain and total Panx1, as expected (Fig. 6a). A high co-localization between Panx1 and DHPR was also observed, as demonstrated by the Manders’ coefficient value of 0.8 (M1, DHPR/Panx1) and 0.5 (M2, Panx1/DHPR) (Fig. 6b). Co-localization between Panx1 and Cav3 was also demonstrated in L6-Panx1 cells (Fig. 6c). All the protein complex constituents assessed co-immunoprecipitated in L6-Panx1 cells: DHPR, Panx1, P2Y_2_R, Dys, and Cav3 (Fig. 6d). For strong evidence of protein-protein proximity in intact L6-Panx1 cells, in situ PLA was assessed. High PLA labeling, depicted as a large amount of red puncta, was detected for DHPR and Panx1 (Fig. 7a), DHPR and Cav3 (Fig. 7b), P2Y_2_R and DHPR (Fig. 7c), Panx1 and Cav3 (Fig. 7d), and P2Y_2_R and Cav3 (Fig. 7e). No PLA staining was observed between DHPR and the nuclear envelope protein Lap2, as expected (Fig. 7f). The cells assessed by in situ PLA were parallel stained with anti-Strep antibodies, to confirm Panx1-Strep tag overexpression (Fig. 7, green panels).

All these results confirm that a multiprotein complex containing DHPR, Panx1, P2Y_2_R, Cav3, and Dys is properly assembled in L6-Panx1 cells.

### Panx1 overexpression increased ATP release and upregulated expression of genes known to be modulated through excitation-transcription coupling

**Fig. 8:**
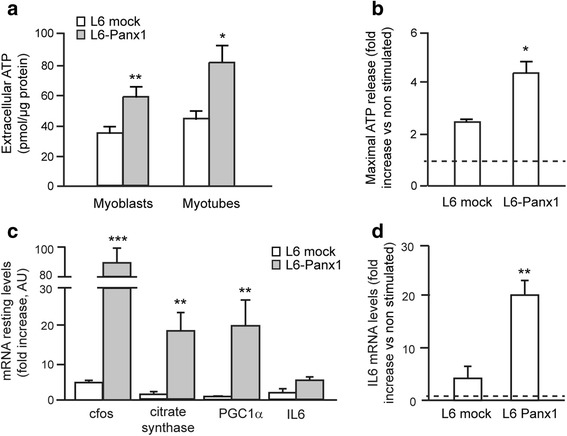
Panx1 overexpression increases resting levels of extracellular ATP and gene expression in muscle L6 cells. **a** Extracellular ATP resting levels are increased in L6 muscle cells overexpressing Panx1 (L6-Panx1), both in myoblast and in myotube differentiation stages (*n* = 3). **b** ATP release evoked by electrical stimulation (20 Hz, 270 pulses, 0.3 ms each) is increased in Panx1 overexpressing cells. ATP release induced by ES is graphed as fold increase compared to non-stimulated cells (*n* = 3). **c** mRNA resting levels of E-T coupling related genes (*cfos,* citrate synthase, PGC1α, and interleukin 6) in either mock or Panx1-overexpressing L6 myotubes,were analyzed by qPCR (*n* = 3-4). **d** Interleukin 6 mRNA levels evoked by ES in both mock and Panx1-overexpressing L6 myotubes were analyzed by qPCR (*n* = 3–4). GAPDH was used as a housekeeper gene for normalization. The *dashed line* (**b**–**d**) marks the value of “1” to indicate the level of expression of IL6 in cells without ES, against which the results are normalized. Values are expressed as mean ± SEM. *p < 0.05; **p < 0.01; ***p < 0.001. All the assays were performed after overnight 5 mM sodium butyrate incubation, to improve Panx1 overexpression as detailed in Fig. 5

We have previously demonstrated that Panx1 is the ATP-release conduit to the extracellular medium in skeletal muscle cells [19, 20]. In order to assess possible physiological alterations evoked by Panx1 overexpression in L6 cells, we measured basal extracellular ATP levels in L6 mock and L6-Panx1 (Fig. 8a). Resting levels of extracellular ATP increased by 60 % after Panx1 overexpression induced with sodium butyrate, both in the myoblast and myotube stages (Fig. 8a). ATP release evoked by membrane depolarization (20-Hz electrical stimulation, 270 pulses, 0.3 ms each) was also significantly increased in L6 myotubes overexpressing Panx1 (Fig. 8b).

We have previously demonstrated that ATP released from skeletal muscle cells through Panx1 channels is relevant for control of gene expression [19, 20, 22]. In order to determine the effect of Panx1 overexpression on skeletal myotube gene expression, mRNA level of selected genes was assessed. *c-fos* was evaluated as an immediate early gene, IL6 was studied because it is a relevant myokine released by muscle activity, and PGC1α and citrate synthase were chosen because of their relevance in mitochondria biogenesis and function. Panx1 overexpression significantly increased resting mRNA level of all tested genes (Fig. 8c), except for IL6. Surprisingly, *c-fos*, citrate synthase (CS) and PGC1α mRNA levels increased 15–20-fold in Panx1 overexpressing cells comparing to the mock cells. Although resting levels of IL6 mRNA did not increase with Panx1 overexpression, the increase in IL6 mRNA evoked by 20-Hz electrical stimulation was upregulated 4.5-fold in L6-Panx1 cells as compared to L6 mock (Fig. 8d).

All these results confirm that Panx1 expression levels modify muscle cell gene expression pattern both at rest and after electrical stimulation, probably through a fine-tuned control of the ATP release.

## Discussion

**Fig. 9:**
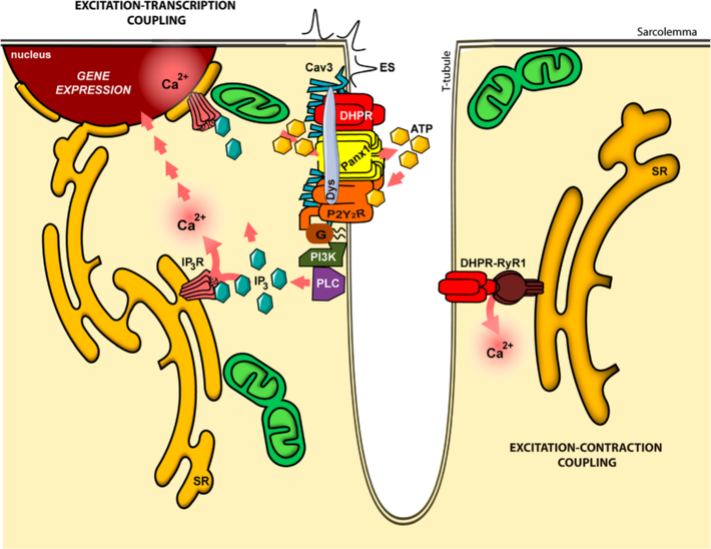
Hypothetical model of the multiprotein complex related to the excitation-transcription coupling in skeletal muscle. This model summarizes the findings discussed in this work and in our previous publications [9, 14, 17, 19, 20]. A multiprotein complex is assembled at the beginning of the T-tubule membrane, including dihydropyridine receptor (DHPR, Ca_v_1.1) as the voltage sensor, pannexin 1 (Panx1) as the ATP release channel, the purinergic metabotropic P2Y_2_ receptor (P2Y_2_R), and caveolin-3 (Cav3), and dystrophin (Dys) as the scaffold proteins. The membrane depolarization sensed by DHPR induce the ATP release through Panx1. The heterotrimeric G protein attached to P2Y_2_R sequentially activate, upon ATP binding, phosphoinositide-3 kinase (PI3K) and phospholipase-C (PLC) to produce IP_3_ and activates calcium release through IP_3_R, for the slow calcium transient. This IP_3_-dependent slow calcium transient is involved in the gene expression control of several genes by the excitation-transcription coupling mechanism. The ryanodine receptor-1 (Ryr1), that is known to interact with DHPR, is involved in the excitation-contraction coupling, related with the fast calcium transients, but whether this protein is part of the same multiprotein complex involved in the excitation-transcription coupling has not yet been elucidated

We have presented evidence showing that Panx1, DHPR, P2Y_2_R, and Cav3 assemble a multiprotein complex in the T-tubule of the skeletal muscle, which is involved in E-T coupling. Previous work in our laboratory demonstrated that extracellular ATP is a key mediator between membrane depolarization and gene expression, leading to skeletal muscle plasticity [19, 21]. This signaling requires the proper coordination between several proteins for the control of gene expression evoked by sarcolemma depolarization, which is consistent with our biochemical (Fig. 2) and cell imaging (Figs. 3 and 4) approach, showing a well-defined protein association among the molecular actors of the E-T coupling process. We have also identified Dys as a part of this multiprotein complex, suggesting a potential role for E-T coupling in muscular pathologies. In this work, we demonstrated that overexpression of one interactor of this multiprotein complex (Panx1) leads to an increased ATP release and altered resting levels of gene expression, reinforcing the relevance of a fine-tune regulation of this complex for skeletal muscle physiology. A hypothetical model that gathers our results is depicted in Fig. 9.

Several signaling complexes have been described in cardiac and skeletal muscles. In skeletal muscle, a macromolecular E-C coupling complex involves RyR, Ca_v_1.1 channel α1S-subunit, calmodulin, calstabin, AKAP, PKA, CaMKII, sorcin, calsequestrin, triadin, and junctin (reviewed in [32–34]). A multiprotein complex between the store operated calcium channel, TRPC1, Dys and syntrophin has been also postulated, and its disassembly has been proposed as a putative explanation for calcium alterations observed in dystrophic muscle cells [35]. In the present work, we demonstrated that an E-T coupling multiprotein complex is also assembled in skeletal muscle.

Co-immunoprecipitation assays suggested interaction between DHPR, Panx1, P2Y_2_R, Cav3, and Dys in skeletal muscle models at different differentiation stages: triads derived from adult mouse skeletal muscles (Fig. 1b), newborn-derived rat myotubes (Fig. 1c), and myotubes derived from the L6 cell line overexpressing Panx1 (Fig. 6d). Multiprotein complex assembly was also demonstrated by blue native SDS-PAGE (Fig. 2). Co-localization of all these proteins was also demonstrated by indirect immunofluorescence and confocal microscopy, both in adult isolated fibers (Fig. 3 and Manders’ coefficients detailed in results section) and in L6-Panx1 cells (Fig. 6a–c), where Manders’ coefficients between 0.4 and 0.8 were observed. But undoubtedly, our main evidence of protein-protein proximity are PLA experiments, indicating that Panx1, DHPR, P2Y_2_R, and Cav3 are located within a distance of less than 40 nm of each other in both intact muscle fibers (Fig. 4) and L6-Panx1 myotubes (Fig. 7).

It is noteworthy that this complex is present in a muscle cell line as L6, even in relatively undifferentiated stages, which suggest a role of this complex in early stages of myogenesis and possibly playing an additional role in the differentiation process to the adult muscle fiber, where it will have a role in muscle plasticity. It is probable that this complex appears previous to the assembly of the E-C coupling machinery in cells that are unable to contract and remains present in fully differentiated skeletal fibers. It has been described that Panx1 plays a role into the muscle differentiation process [43]. Although Panx1 is expressed throughout the proliferation and differentiation of muscle cells, its overexpression has been described during the differentiation to myotubes in vitro, which is correlated with our results in the L6-Panx1 cell line. Langlois et al. proposed that Panx1 play a role in the ATP release and Ca^+2^ signaling required for the proper myogenesis, which opens the possibility that the E-T coupling complex could be also playing an important role in this process.

In this work, we also demonstrated the interaction of Dys with the E-T coupling machinery. Using a co-immunoprecipitation approach, we identified the interaction of Dys with Panx1, DHPR, and P2Y_2_R (Fig. 1). The presence of Dys in the multiprotein complex suggests a role for the E-T coupling mechanism in muscular dystrophies, as Duchenne muscular dystrophy (DMD). We have previously reported that skeletal fibers from a mouse model of DMD (*mdx* mouse) have increased resting levels of extracellular ATP, but this release turns independent on membrane depolarization [44]. In *mdx* skeletal muscle triads, there are higher levels of Panx1 but lower levels of Ca_v_1.1, and their association demonstrated by co-immunoprecipitation is significantly reduced, suggesting a disruption of the E-T coupling multiprotein complex produced by the absence of Dys. In *mdx* muscle fibers, gene expression induced by addition of extracellular ATP appears completely altered; while exogenous ATP is antiapoptotic for normal skeletal fibers, it activates pro-apoptotic pathways in fibers derived from *mdx* mice. The latter, together with the increased resting levels of ATP, could account for the exacerbated apoptosis in *mdx* muscles [44]. We have also reported that DHPR blockade by daily injections of nifedipine over 1 week reduced resting levels of ATP in *mdx* skeletal fibers, as well as pro-apoptotic genes expression. Interestingly, DHPR blockade improves muscle strength and exercise tolerance in *mdx* mice. These results suggest that nifedipine reduces basal ATP release, thereby decreasing purinergic receptor activation, which in turn reduces resting Ca^2+^ levels in mdx skeletal muscle cells [45]. A putative interaction between Ca_v_1.1 and Dys in both normal and DMD muscles has also been described [46], and also a DHPR malfunction was described in DMD muscle fibers [47]. All this background and our new results suggest that Dys is a relevant interactor for the stability of the E-T coupling multiprotein complex, leading to dramatic physiological alterations when absent.

The existence of the DHPR in both E-C and E-T complexes opens the question if there is a unique extra-large multiprotein complex involved in both processes or if there are two pools of DHPR forming two different multiprotein complexes. We did not directly address this point in this work. However, we have results of blue native SDS-PAGE in mouse triads that show a distribution of RyR1 that co-localizes with DHPR in vertical lines different to the ones where DHPR co-localizes with Panx1 (not shown), suggesting the presence of independent complexes. This possibility is reinforced considering the very restricted location of Panx1 and Cav3 in a specific T-tubule zone near the surface, while DHPR is widely expressed along the T-tubule (Fig. 3), as Ryr1 does at the sarcoplasmic reticulum all along T-tubule proximity [48, 49]. The analysis of the z-projection of the whole fiber for Panx1 and Cav3 (Fig. 3b–d) shows a maximal expression in a zone of the T-tubule invagination near the surface of the fiber (3–5-μm depth). Even more, the analysis of the positive PLA dot distribution at the medial plane of skeletal fibers showed that interaction of DHPR/Panx1, DHPR/Cav3, and Panx1/Cav3 occurs in a restricted region near the sarcolemma, without entering the depth of the fiber (Fig. 4b). In this initial portion of the T-tubule, the presence of caveolae has been described [50], which is consistent with our results for the presence of Cav3 in the multiprotein complex. The assembly of the multiprotein complex in this particular area suggests that the E-T coupling could occur in a zone near the nuclei and sub-sarcolemmal mitochondria in the muscle fiber, in an optimal environment for coordinating ATP production and release with signal transduction and gene expression [51].

Interestingly, unlike Cav3 and Panx1, we find that P2Y_2_R is located throughout the depth of the fiber, as does DHPR (Fig. 3e). Moreover, interaction between P2Y_2_R and DHPR also occurs across the entire area of the fiber, as shown by the analysis of PLA of a medial plane of the fiber (Fig. 4b). This finding suggests that P2Y_2_R and DHPR assemble a different complex at the T-tubule depth than the one assembled at the entrance with Panx1 and Cav3, implicated in different physiological events.

To address the physiological relevance of the proposed multiprotein complex, we studied putative alterations when one interactor is overexpressed. To resemble the Panx1 overexpression detected in dystrophic *mdx* cells, we developed a variant of the rat muscle L6 cell line that stably overexpress Panx1 linked to a Tween-Strep-tag. This cell line properly assembled the multiprotein complex containing the DHPR, Panx1, P2Y_2_R, Cav3, and Dys, as demonstrated by co-immunoprecipitation, confocal microscopy, and PLA assays (Figs. 6 and 7). L6 cells overexpressing Panx1 showed significantly increased levels of extracellular ATP at rest, reinforcing our previous demonstration that Panx1 is the conduit for ATP release in skeletal muscle cells [19, 20]. ATP release evoked by electrical stimulation showed a 70 % increase in L6-Panx1 cells. In basal conditions, expression of genes previously related to the ET coupling process and muscle plasticity was addressed. We observed near 18-fold increase in mRNA levels for the early gene cfos in L6-Panx1 cells (Fig. 8c) [14, 20]. We also found that L6-Panx1 cells that release higher levels of ATP to the extracellular medium have 15–20-fold increase in the expression of the oxidative markers citrate synthase and PGC1α at rest, resembling a slow fiber expression pattern (Fig. 8c). Interestingly, although we have previously described that IL6 is a myokine strongly regulated through extracellular ATP for its expression and secretion [22], we have not found significant differences in resting levels of IL6 mRNA when overexpressing Panx1 (Fig. 8c). However, IL6 expression evoked by 20-Hz electrical stimulation (ES) was strongly upregulated in L6-Panx1 cells (Fig. 8d). One tentative explanation for this is that L6 cells may require higher levels of extracellular ATP to promote IL6 expression or additional signaling events related to membrane depolarization besides extracellular ATP.

In contradistinction to that described in mdx cells, overexpression of Panx1 in L6 cells did not modify DHPR expression levels or DHPR/Panx1 interaction, and cell phenotype and viability were similar than L6 mock. A putative explanation is that, for a proper role, not only Panx1 expression levels are required to be normal but also the proper assembly of the E-T complex. That step could be mostly altered in dystrophic cells due to dystrophin deficiency.

There is increasing evidence pointing that purinergic signaling in adult skeletal muscle fibers goes beyond regulation of gene expression; extracellular ATP is capable of regulating glucose transport and possibly other aspects of metabolism [52]. On the other hand, extracellular ATP can regulate activation of NOX2 and ROS production in the muscle fiber, acting as an important pathway to regulate the redox state of the muscle [53]. All this evidence points to a central role of the so cold E-T complex as an essential structure linking the electrical activity of the muscle fiber with its adaptation to the requirements that such activity poses in terms of changes in metabolism and plasticity [21]. We are aware that in the present work, we describe the interaction of several molecules but need to know the physiological relevance of the presence or absence of each of them into the E-T coupling complex and their implication in skeletal muscle plasticity. Knowing the fine tuning between complex proteins interaction and expression in health and disease, or during processes as development or aging, is our next challenge.

## Conclusions

- In the present work, we demonstrate the existence of a multiprotein complex related to the E-T coupling process, which harbors the voltage sensor DHPR and proteins related to ATP release (Panx1 channel) and extracellular ATP signaling (P2Y_2_R). Considering that we have previously reported extracellular ATP as a relevant mediator between membrane depolarization and gene expression involved in E-T coupling, the present work suggests the requirement of a close association of all the signaling interactors for a fine-tuned regulation of muscle signaling and plasticity. The next logical step, which we are already working on, is to determine if physical interaction is required to coordinate these proteins function.
- Dys and Cav3, two proteins with relevant roles in scaffolding and signaling in skeletal muscle, were also detected as multiprotein complex interactors. The presence of Dys suggests a relevant role of this multiprotein complex in muscular pathologies; we have previously described a dysregulation of this complex expression and function in a dystrophic cell line lacking Dys expression.
- By immunofluorescence, proximity ligation assays, and advanced confocal microscopy z-reconstructions, we demonstrated that the multiprotein complex is located at the T-tubules (due to the DHPR known location), in a very restricted region near to the sarcolemma (3–5-μm depth). This initial portion of the T-tubule has been previously described as enriched in caveolae. All these evidences suggest that the multiprotein complex of DHPR with proteins involved in E-T coupling could be spatially separated from the E-C coupling complex with Ryr1 that runs all along the T-tubule depth.
- Overexpression of one of the multiprotein complex interactors (Panx1) potentiates both ATP release and gene expression, both at rest and after electrical stimulation, suggesting that this complex is an interesting target to treat muscle plasticity disorders and to improve muscle performance. How this complex structure and function behaves in health and disease, or during muscle development and aging, are interesting points to be addressed in the future.

## Ethics approval and consent to participate

All protocols of Animal Handling were approved by the Bioethics Committee, Faculty of Medicine, Universidad de Chile.

## Consent for publication

Not applicable.

## Abbreviations

ATP: adenosine 5′-triphosphate
Cav3: caveolin-3
CS: citrate synthase
DHPR: dihydropyridine receptor
Dys: dystrophin
E-C: excitation-contraction
ES: electrical stimulation
E-T: excitation-transcription
FDB: *flexor digitorium brevis*
GAPDH: glyceraldehyde 3-phosphate dehydrogenase
IP_3_: inositol 1,4,5-triphospate
P2Y_2_R: P2Y_2_ receptor
Panx1: pannexin 1
PGC1α: peroxisome proliferator-activated receptor gamma coactivator 1-alpha
PLA: proximity ligation assay
T: transverse
